# Influence of a Decaying Cyclonic Eddy on Biogenic Silica and Particulate Organic Carbon in the Tropical South China Sea Based on ^234^Th-^238^U Disequilibrium

**DOI:** 10.1371/journal.pone.0136948

**Published:** 2015-08-28

**Authors:** Weifeng Yang, Min Chen, Minfang Zheng, Zhigang He, Xinxing Zhang, Yusheng Qiu, Wangbin Xu, Lili Ma, Zhiyu Lin, Wangjiang Hu, Jian Zeng

**Affiliations:** 1 State Key Laboratory of Marine Environmental Science, Xiamen University, Xiamen, China; 2 College of Ocean and Earth Sciences, Xiamen University, Xiamen, China; Glasgow University, UNITED KINGDOM

## Abstract

Eddies play a critical role in regulating the biological pump by pumping new nutrients to the euphotic zone. However, the effects of cyclonic eddies on particle export are not well understood. Here, biogenic silica (BSi) and particulate organic carbon (POC) exports were examined inside and outside a decaying cyclonic eddy using ^234^Th-^238^U disequilibria in the tropical South China Sea. For the eddy and outside stations, the average concentrations of BSi in the euphotic zone were 0.17±0.09 μmol L^-1^ (mean±sd, n = 20) and 0.21±0.06 μmol L^-1^ (n = 34). The POC concentrations were 1.42±0.56 μmol L^-1^ (n = 34) and 1.30±0.46 μmol L^-1^ (n = 51). Both BSi and POC abundances did not show change at the 95% confidence level. Based on the ^234^Th-^238^U model, BSi export fluxes in the eddy averaged 0.18±0.15 mmol Si m^-2^ d^-1^, which was comparable with the 0.40±0.20 mmol Si m^-2^ d^-1^ outside the eddy. Similarly, the average POC export fluxes were 1.5±1.4 mmol C m^-2^ d^-1^ and 1.9±1.3 mmol C m^-2^ d^-1^ for the eddy and outside stations. From these results we concluded that cyclonic eddies in their decaying phase have little effect on the abundance and export of biogenic particles.

## Introduction

Mesoscale eddies significantly affect biogeochemical processes in the upper ocean especially in oligotrophic oceanic settings [1–4]. Three types of eddies, i.e., cyclonic, anticyclonic and mode-water eddies, are reported [5], all of which supply new nutrients to the euphotic zone either through uplift of isopycnals [6] or through horizontal advection between the eddy center and edge [7]. Commonly, cyclonic eddy-induced nutrients stimulate phytoplankton growth, the production of particulate organic carbon (POC) and biogenic silica (BSi) at the early life-stage of the eddy [8–9]. However, POC export shows little variation although an increase of BSi export is commonly observed [3–4, 10–11]. In these case studies, diatom groups make up the majority of the phytoplankton biomass [4, 12]. It is assumed that diatom-contained POC is probably grazed by large ciliates and heterotrophic dinoflagellates [13], and empty diatom frustules remain [14]. Bacterial activity is also hypothesized to increase POC remineralization and decrease POC export [15]. Consequently, particles leaving the euphotic zone are Si-rich but C-poor [10]. At the same time, negative net community production (NCP) rates are observed in older cyclones [16], indicating that the life-stage of the eddy might be important to regulate the production and export of biogenic particles in the eddy. Thus, more investigations are needed to improve our understanding of the highly temporal variability in particle dynamics in both cyclonic and anticyclonic eddies especially at different life-stages [7, 17].

The South China Sea (SCS) is the largest marginal sea in the western Pacific Ocean. Owing to its significance to our understanding of marine biogeochemistry, the SCS has become an important deep-water research location. More than 30 eddies are observed each year in the SCS [18–19]. However, the interactions between the mesoscale eddy and biogenic particle export in the SCS are poorly understood [7]. Our study examined the influence of a cyclonic eddy, at its decaying stage, on the abundances of POC and BSi and their export in the tropical SCS.

## Sampling and Methods

### Ethics Statement

No specific permits were required for this study and the locations are not privately owned. The South China Sea Institute of Oceanology and The Chinese Academy of Sciences issued permission for each location. The field studies did not involve endangered or protected species.

### Study area

The SCS is located in Southeast Asia, with a shallow mixed layer of <50 m throughout the year. The euphotic zone of >1% light is usually 75–100 m [20], resulting in a rapid consumption of nutrients in the upper 100 m. Hence, the SCS shows typical oligotrophic characteristics [21] similar to the Sargasso Sea and the North Pacific gyre. To date, very limited nutrients have been reported in the SCS basin, especially in the southern SCS. The primary production (PP) is usually less than 46 mmol C m^-2^ d^-1^ [22] with high values in seasons showing a deeper mixed layer [23]. Model simulations indicate that physical processes, such as eddies and monsoon-forced circulation, to a large degree, affect PP in the SCS [19–20, 24]. However, the response of biogenic particles (including their abundance in and export out of the euphotic zone) to these physical processes has been poorly understood in the SCS. The *in situ* investigation identified involves only the influence of an anticyclonic eddy on the export of POC and BSi [7], which shows enhancement of POC export, contrary to the general concept.

### Sample collection

**Fig 1:**
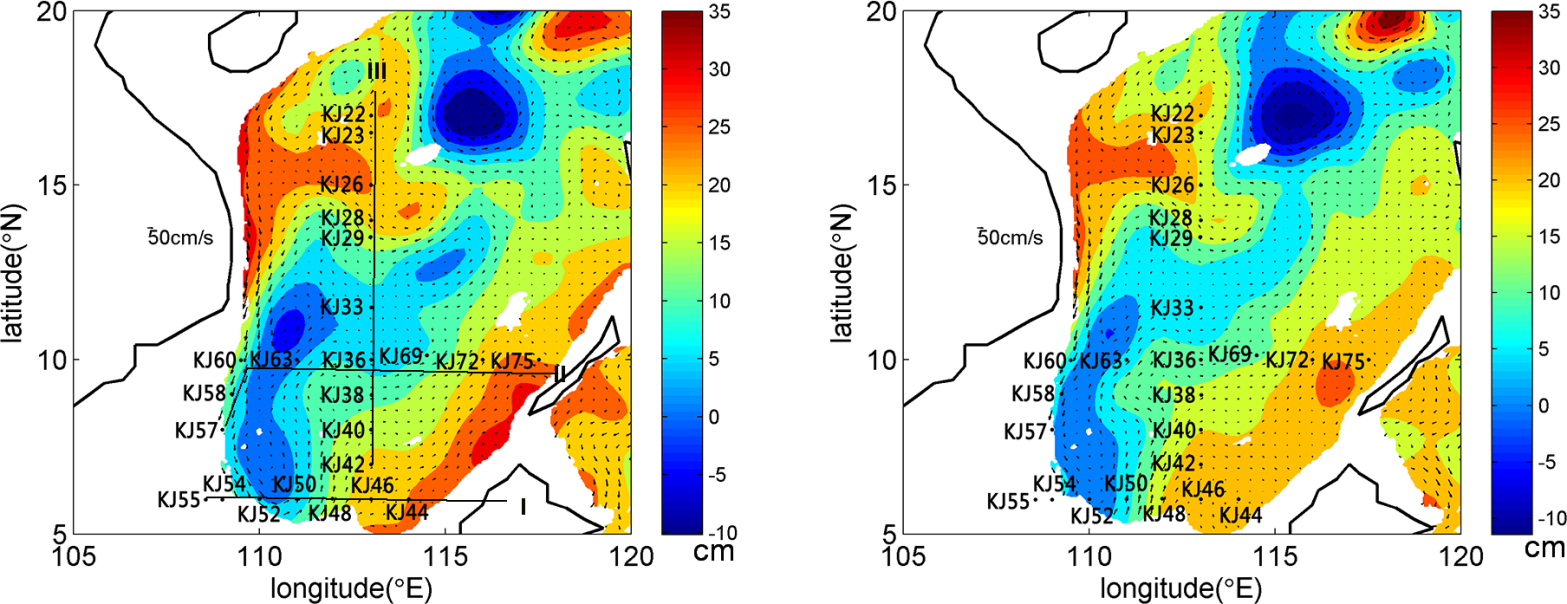
Sampling stations along Transects *I*, *II*, and *III* in the tropical SCS, and the sea-level anomaly (SLA) on 10 November (left) and 17 November (right), 2010. The color scale units are centimeters.

Samples were collected from 7 to 18 November 2010 on board R/V *SHIYAN 3*. Stations were situated mainly in the tropical SCS (Fig 1), covering the 6–17°N and 109–118°E area. Transects *I* and *II*, mainly along 6°N and 10°N, involved 15 stations and, in addition, a south-north oriented Transect *III* along 113°E included 10 stations. Seawater samples were collected mainly in the euphotic zone (normally ~1, 25, 50, 75, and 100 m) using CTD (conductivity, temperature and depth) Rosette integrated sampling bottles. Generally, 6–8 L of seawater was filtered through a pre-combusted (450°C for 4 h) quartz fiber filter with 1 μm pore-size (QMA, Whatman) to collect particles for POC and particulate ^234^Th measurements. Four liters of filtrate was used to determine the dissolved ^234^Th. BSi samples were collected only from 15 selected stations. Usually, 2 L of seawater was filtered through a 0.8 μm polycarbonate membrane filter to concentrate the BSi.

### ^234^Th analyses

^234^Th in the <1 μm fraction (i.e. dissolved ^234^Th) was co-precipitated using the MnO_2_ method [25]. In order to obtain a stable ^234^Th recovery, we examined the co-precipitation conditions in detail, including filter membrane, pH value and amount of MnO_2_, using calibrated Th yield tracers [26]. We found a mean recovery of ^234^Th of 95.7±1.0% (mean±sd, n = 5). In this study, dissolved ^234^Th was concentrated using co-precipitation conditions [26]. The pH value of the filtrate was adjusted to 9.0 and a solution containing KMnO_4_ and MnCl_2_ was added quantitatively while stirring. The resulting suspension containing precipitated MnO_2_ was left to stand for more than 6 h to allow the MnO_2_ particles to grow, since large particles benefit filtration [27]. MnO_2_ carried ^234^Th was finally filtered on a QMA membrane filter and dried at 60°C. All the dried samples were counted using a low-level beta counter with a counting efficiency of 41.2% until the net counting errors were less than ±5%. 150 days later, a second counting was conducted for quantifying other beta emitters in order to remove their contribution to the first obtained counters [25]. The activity concentrations of ^234^Th were calculated based on the counting efficiency, recovery of Th, and net ^234^Th counts (excluding blank and background), and corrected to sampling time (Table 1). The errors for the ^234^Th data in Table 1 represent the propagated errors from the statistical count errors of ^234^Th obtained from two measurements. ^238^U activities were calculated from salinity based on the widely used relationship between ^238^U (dpm L^-1^) and salinity from the dataset [28]. Salinity was measured with the calibrated CTD.

**Table 1:** Temperature, salinity, activity concentrations of ^234^Th and ^238^U, POC and BSi concentrations, and the δ^13^C value of POC in the SCS in November, 2010. The errors of the ^234^Th datasets represent the propagated errors from the statistical count errors of ^234^Th based on two measurements.

Stations and depths	T	S	^234^Th_P_	^234^Th_D_	^234^Th_T_	^238^U	BSi	POC	δ^13^C
(m)	(°C)		(dpm L^-1^)				(μmol L^-1^)		(‰)
KJ2217.00°N, 113.00°E									
2	28.173	33.591	0.44±0.05	1.86±0.16	2.30±0.17	2.38	-	1.6	-24.32
25	28.206	33.587	0.62±0.05	1.56±0.15	2.17±0.16	2.38	-	1.3	-23.72
50	27.175	33.703	0.63±0.05	1.73±0.16	2.36±0.17	2.39	-	1.5	-23.40
75	24.807	33.755	0.57±0.05	1.87±0.17	2.45±0.18	2.39	-	0.7	-24.60
100	21.701	34.301	0.61±0.06	2.11±0.18	2.72±0.19	2.43	-	0.6	-23.43
200	14.984	34.531	0.56±0.05	1.71±0.16	2.27±0.16	2.45	-	0.8	-22.73
KJ2316.50°N, 113.00°E									
2	28.147	33.482	0.38±0.04	1.69±0.15	2.07±0.16	2.37	-	1.3	-24.66
25	28.156	33.510	0.47±0.05	2.17±0.17	2.64±0.18	2.37	-	1.1	-23.26
50	27.066	33.700	0.36±0.04	1.65±0.15	2.01±0.15	2.39	-	1.1	-23.00
75	25.705	33.760	0.17±0.03	2.00±0.18	2.17±0.18	2.39	-	0.7	-23.51
100	19.657	33.874	0.56±0.05	2.25±0.18	2.81±0.19	2.40	-	0.5	-23.54
200	11.811	34.451	0.68±0.06	1.18±0.13	1.87±0.14	2.44	-	0.4	-23.49
KJ2615.00°N, 113.00°E									
0	-	33.290	0.44±0.04	1.79±0.18	2.23±0.18	2.36	0.12	1.4	-24.25
KJ2814.00°N, 113.00°E									
0	28.893	33.292	0.72±0.06	1.96±0.17	2.69±0.18	2.36	0.18	1.1	-23.84
KJ2913.51°N, 112.99°E									
0	29.004	33.375	0.70±0.07	1.19±0.14	1.88±0.16	2.36	-	0.7	-22.33
KJ3311.50°N, 113.00°E									
0	28.986	32.239	0.34±0.04	2.11±0.18	2.45±0.19	2.28	0.15	1.2	-22.14
25	25.792	33.628	0.56±0.06	1.68±0.15	2.24±0.16	2.38	-	1.0	-22.45
50	22.193	33.900	0.42±0.05	1.38±0.15	1.80±0.15	2.40	-	1.2	-23.16
75	21.576	34.200	0.57±0.06	2.05±0.18	2.62±0.19	2.42	-	1.1	-22.92
100	18.926	34.300	0.34±0.04	2.19±0.19	2.53±0.19	2.43	-	0.7	-24.40
200	14.188	34.518	0.28±0.04	1.47±0.15	1.74±0.15	2.44	-	0.4	-22.66
KJ388.99°N, 113.02°E									
0	-	32.800	0.47±0.05	1.73±0.15	2.19±0.16	2.32	0.18	-	-
KJ408.00°N, 113.00°E									
0	29.348	32.807	0.98±0.08	1.22±0.14	2.20±0.16	2.32	0.23	0.7	-21.66
25	29.436	33.112	0.37±0.05	1.16±0.14	1.53±0.15	2.34	0.14	0.8	-23.81
50	29.299	33.145	1.10±0.09	0.65±0.11	1.75±0.14	2.35	0.20	0.7	-22.13
75	25.98	34.418	0.43±0.05	1.02±0.13	1.44±0.14	2.44	0.30	0.8	-23.79
100	20.213	34.470	0.57±0.06	1.54±0.16	2.10±0.17	2.44	0.15	0.5	-24.42
200	15.372	34.524	0.44±0.05	1.44±0.15	1.88±0.16	2.44	0.13	0.4	-23.18
KJ427.00°N, 113.00°E									
0	29.507	32.397	0.52±0.05	1.12±0.12	1.63±0.13	2.29	0.20	1.0	-23.23
25	29.315	32.647	0.64±0.06	1.73±0.16	2.37±0.17	2.31	0.19	0.8	-22.01
50	29.366	33.115	1.14±0.07	0.99±0.12	2.13±0.14	2.34	0.22	0.8	-22.13
75	25.843	34.012	0.78±0.07	1.01±0.12	1.79±0.14	2.41	0.21	0.9	-24.12
100	19.697	34.260	0.25±0.04	1.22±0.14	1.47±0.14	2.43	0.14	0.7	-25.00
200	16.793	34.505	0.56±0.06	1.28±0.14	1.83±0.15	2.44	0.08	0.5	-23.86
KJ446.00°N, 114.02°E									
0	29.373	32.400	0.79±0.06	1.43±0.14	2.22±0.15	2.29	0.31	1.6	-23.80
25	28.652	32.500	0.67±0.06	0.63±0.09	1.31±0.11	2.30	0.24	0.9	-22.73
50	25.524	33.000	0.56±0.05	1.76±0.15	2.32±0.16	2.34	0.19	0.9	-23.01
75	27.115	33.950	0.52±0.05	0.99±0.12	1.50±0.13	2.40	0.16	1.1	-23.48
100	23.390	34.300	0.72±0.06	1.15±0.12	1.87±0.14	2.43	0.27	0.6	-24.13
200	16.623	34.522	0.53±0.05	1.43±0.14	1.96±0.15	2.44	0.10	0.5	-22.54
KJ466.00°N, 113.00°E									
0	29.756	32.379	0.54±0.06	1.14±0.14	1.68±0.15	2.29	0.24	1.0	-22.76
25	29.405	32.428	0.37±0.05	1.14±0.14	1.51±0.15	2.30	0.25	1.5	-22.21
50	29.391	32.962	1.18±0.09	1.36±0.15	2.53±0.17	2.33	0.27	1.4	-22.78
75	26.464	33.977	0.10±0.03	1.68±0.17	1.78±0.17	2.41	0.26	1.6	-23.90
100	21.947	34.209	1.29±0.09	1.18±0.14	2.48±0.17	2.42	0.16	1.0	-24.22
200	17.086	34.512	1.22±0.09	0.57±0.1	1.78±0.13	2.44	0.25	1.0	-23.33
KJ486.01°N, 111.97°E									
0	28.971	32.500	1.20±0.10	0.74±0.12	1.94±0.15	2.30	0.21	-	-
25	28.857	32.504	0.08±0.02	2.24±0.20	2.31±0.20	2.30	0.16	-	-
50	26.305	33.596	0.96±0.08	0.93±0.13	1.89±0.16	2.38	0.26	2.3	-22.68
75	22.200	34.208	0.74±0.08	0.04±0.03	0.78±0.08	2.42	0.21	1.5	-25.04
100	21.448	34.300	0.41±0.06	1.41±0.17	1.82±0.18	2.43	0.12	1.5	-24.29
200	18.917	34.385	0.90±0.08	0.85±0.13	1.75±0.16	2.43	0.04	1.0	-23.16
KJ506.00°N, 111.00°E									
0	29.150	32.601	1.27±0.10	0.32±0.07	1.59±0.12	2.31	0.14	1.3	-22.18
25	29.180	32.982	0.65±0.07	1.25±0.14	1.90±0.16	2.34	0.16	1.3	-21.73
50	26.060	34.397	0.11±0.03	0.96±0.12	1.07±0.12	2.44	0.17	1.6	-22.93
75	21.574	34.315	1.25±0.09	1.12±0.13	2.37±0.16	2.43	0.09	-	-
100	19.946	34.420	0.91±0.08	0.94±0.12	1.85±0.14	2.44	0.09	0.5	-23.16
200	15.886	34.517	0.09±0.02	1.79±0.17	1.88±0.17	2.44	0.12	0.6	-23.19
KJ526.00°N, 110.00°E									
0	29.300	32.845	1.32±0.10	0.91±0.14	2.23±0.17	2.33	0.12	1.5	-21.14
25	29.158	32.929	0.41±0.06	1.68±0.18	2.10±0.19	2.33	0.19	1.3	-21.22
50	26.264	33.920	1.39±0.10	0.91±0.13	2.30±0.17	2.40	0.27	2.8	-23.70
75	22.394	34.321	1.31±0.10	0.76±0.12	2.07±0.16	2.43	0.22	0.7	-24.62
100	20.189	34.383	0.67±0.07	0.81±0.13	1.47±0.14	2.43	0.15	0.6	-23.41
150	18.260	34.476	0.16±0.03	1.47±0.17	1.63±0.17	2.44	0.11	0.5	-22.71
KJ545.99°N, 109.00°E									
0	-	32.910	1.53±0.12	0.32±0.08	1.85±0.14	2.33	0.11	1.9	-22.76
25	28.364	33.159	1.68±0.12	0.84±0.14	2.52±0.18	2.35	0.15	1.6	-21.79
50	25.255	33.936	0.41±0.06	0.96±0.15	1.37±0.16	2.40	0.26	1.9	-23.26
75	22.874	34.188	0.46±0.06	0.28±0.07	0.74±0.09	2.42	0.10	1.1	-23.10
100	22.245	34.248	0.88±0.09	1.20±0.16	2.08±0.19	2.43	0.08	1.0	-22.96
KJ555.99°N, 108.56°E									
0	28.642	32.990	0.84±0.08	0.91±0.13	1.75±0.15	2.34	0.23	2.3	-21.53
25	25.750	33.516	0.50±0.07	1.49±0.17	1.99±0.18	2.37	0.32	1.9	-21.89
50	25.612	33.575	0.47±0.06	1.19±0.15	1.66±0.16	2.38	0.25	1.4	-21.95
75	25.502	33.618	0.51±0.06	0.38±0.08	0.89±0.10	2.38	0.24	1.3	-22.30
KJ577.99°N, 109.00°E									
0	-	34.100	0.45±0.06	0.86±0.13	1.31±0.15	2.41	0.36	1.7	-22.31
25	27.633	34.105	0.61±0.07	0.52±0.11	1.14±0.13	2.42	0.24	1.8	-22.42
50	23.943	34.030	1.61±0.11	1.01±0.15	2.62±0.18	2.41	0.14	1.0	-23.01
75	22.759	34.143	0.53±0.06	1.46±0.18	1.99±0.19	2.42	0.04	1.1	-22.81
100	20.808	34.394	1.51±0.10	1.14±0.16	2.64±0.19	2.44	0.03	2.0	-22.95
KJ589.01°N, 109.25°E									
0	28.417	33.105	0.42±0.06	2.14±0.24	2.56±0.25	2.34	0.23	1.4	-21.94
25	28.276	33.152	0.15±0.04	1.23±0.18	1.39±0.19	2.35	0.17	1.5	-22.34
50	27.540	34.272	0.29±0.05	1.07±0.17	1.36±0.18	2.43	0.35	2.3	-22.95
75	23.713	34.013	0.69±0.08	1.08±0.17	1.77±0.19	2.41	0.16	2.2	-23.67
100	21.877	34.218	0.29±0.05	2.18±0.24	2.46±0.25	2.42	0.07	0.7	-22.82
200	15.213	34.451	0.58±0.07	1.69±0.22	2.27±0.23	2.44	0.15	0.4	-25.15
KJ609.99°N, 109.50°E									
0	-	33.100	0.52±0.06	1.94±0.21	2.46±0.22	2.34	-	2.4	-23.79
25	28.057	33.199	0.17±0.03	1.94±0.21	2.11±0.21	2.35	-	2.0	-22.56
50	26.155	33.409	0.15±0.03	1.82±0.21	1.97±0.21	2.37	-	2.2	-21.71
75	23.616	34.120	0.56±0.06	1.87±0.21	2.43±0.22	2.42	-	1.0	-22.78
100	20.813	34.280	0.55±0.07	2.28±0.23	2.83±0.24	2.43	-	0.9	-23.65
200	15.007	34.451	0.31±0.05	1.76±0.21	2.07±0.21	2.44	-	0.7	-22.54
KJ6310.00°N, 111.00°E									
0	29.201	32.032	0.43±0.04	1.50±0.18	1.93±0.19	2.27	-	1.5	-22.53
25	29.197	32.146	0.78±0.05	0.80±0.14	1.58±0.15	2.28	-	1.6	-22.60
50	28.884	32.146	0.59±0.05	1.38±0.18	1.97±0.18	2.28	-	1.8	-22.81
75	22.610	34.057	0.61±0.05	0.74±0.13	1.36±0.14	2.41	-	1.2	-24.19
100	18.665	34.343	0.55±0.04	2.10±0.22	2.66±0.23	2.43	-	1.1	-23.38
200	14.587	34.445	0.50±0.04	0.70±0.12	1.20±0.13	2.44	-	0.5	-
KJ3610.00°N, 113.01°E									
0	29.078	32.805	0.42±0.07	1.27±0.19	1.70±0.21	2.32	-	1.7	-22.46
25	29.088	32.804	0.25±0.05	2.06±0.24	2.31±0.25	2.32	-	1.2	-21.86
50	27.664	33.644	0.48±0.07	1.33±0.19	1.82±0.20	2.38	-	1.2	-23.04
75	23.125	34.018	0.75±0.09	0.74±0.15	1.50±0.18	2.41	-	1.7	-25.57
100	19.967	34.255	0.90±0.10	1.34±0.19	2.24±0.22	2.43	-	1.5	-24.53
200	16.001	34.459	0.51±0.07	1.00±0.17	1.51±0.18	2.44	-	0.5	-24.15
KJ6910.12°, 114.50°E									
0	29.333	32.887	0.86±0.09	1.15±0.17	2.01±0.20	2.33	-	1.6	-22.73
25	29.063	32.895	0.46±0.07	2.12±0.23	2.58±0.24	2.33	-	2.2	-22.75
50	28.609	33.313	0.29±0.05	1.58±0.20	1.87±0.20	2.36	-	1.5	-22.86
75	24.046	34.110	1.07±0.10	0.79±0.14	1.86±0.17	2.42	-	1.6	-24.97
100	21.074	34.224	0.94±0.10	1.56±0.20	2.49±0.22	2.42	-	0.7	-25.34
200	15.054	34.501	0.23±0.05	1.92±0.23	2.15±0.23	2.44	-	0.7	-24.03
KJ7210.00°N, 116.00°E									
0	29.428	32.729	0.44±0.04	1.72±0.20	2.17±0.21	2.32	-	1.7	-22.23
25	29.501	33.010	0.41±0.08	1.10±0.16	1.51±0.18	2.34	-	1.3	-22.03
50	29.411	33.085	0.57±0.05	1.26±0.17	1.83±0.18	2.34	-	1.3	-22.49
75	26.050	34.057	0.41±0.08	0.97±0.15	1.37±0.17	2.41	-	1.7	-23.44
100	21.821	34.291	0.74±0.05	1.52±0.19	2.26±0.20	2.43	-	0.8	-24.61
200	15.629	34.506	0.42±0.04	1.11±0.16	1.53±0.17	2.44	-	0.5	-24.31
KJ7510.00°N, 117.52°E									
0	29.587	32.766	0.42±0.06	1.95±0.25	2.37±0.25	2.32	-	1.9	-22.93
25	29.446	32.816	0.51±0.07	1.70±0.22	2.21±0.23	2.32	-	1.5	-22.50
50	29.403	32.979	0.40±0.06	0.85±0.15	1.25±0.17	2.34	-	1.3	-22.39
100	24.638	34.180	1.11±0.10	1.54±0.22	2.66±0.24	2.42	-	1.1	-24.15
200	16.094	34.497	0.53±0.07	1.54±0.22	2.07±0.23	2.44	-	0.7	-23.90

### POC and BSi analyses

POC was determined using the particulate ^234^Th sample. Such a strategy can best support the accuracy of the ^234^Th-based POC flux [29]. Reviews indicate that the influence of particle size on the C/Th ratio is complicated [30–31]. Because of limited shiptime, >53 μm particles were not collected in the present study. And >1.0 μm particles were used to quantify the POC flux as previously used in the SCS [7]. The QMA filters were acid-fumigated with concentrated HCl (12 mol L^-1^) for 48 h to remove carbonate. After drying at 60°C, the POC content and the ^13^C abundance in POC (δ^13^C) were determined with a Perkin Elmer CHN analyzer connected to a Finnigan MAT DELTA^plus^ XP mass spectrometer [32]. The standard used for POC and δ^13^C was IAEA-C8. The procedural carbon blank including filter and tin cup was less than 3 μg, accounting for less than 8% of the bulk POC. Based on the replicate analyses, the uncertainties of the POC contents were better than 10%. The double wet-alkaline digestion method [33] was adopted to determine BSi contents. Briefly, particles on polycarbonate membranes were digested with 0.2 mol L^-1^ NaOH. Silica concentration was measured through molybdosilicate blue spectrophotometry. Then particles were rinsed with silica-free water and dried. A second digestion was conducted with the same protocols to quantify the mineral-derived silica [33]. The BSi contents were calculated based on the sequential leaching. The procedural blank of BSi, including regents and membrane, was less than 0.03 μmol L^-1^. Two reference materials (still pond and R64) used for inter-laboratory comparison [34] were used for BSi determination. Our results, 2.79±0.26% (mean±sd) for the still pond sample and 5.27±0.24% for the R64 sample, were comparable with the inter-laboratory averages of 2.82±1.17% and 6.49±2.09%.

## Results

### Sea-level anomaly (SLA)

To examine the possible influence of a cyclonic eddy on biological particle abundance and export, stations in the eddy were separated from those located outside the eddy based on the SLA with weekly resolution obtained from the Global Delayed-Time merged SLA Data (Fig 1). SLA represents the variations of the sea surface height (SSH) relative to a mean sea surface (MSS). The MSS is the mean of the SSH from 1993 to 1999. All samples were collected from 7 to 18 November. Based on the SLA contours on 10 and 17 November, the cyclonic eddy, lying in the southwest part of the sampling area, was at its decaying stage, as was confirmed by the temporal evolution of SLA from 13 October to 1 December, 2010 (not shown). The eddy was elongated extending from 6° to 12°N, and the core located around 11°N, 111°E. The maximum SLA was much lower than 0 cm at the eddy center on 10 November, and the area with SLA decreased on 17 November.

**Fig 2:**
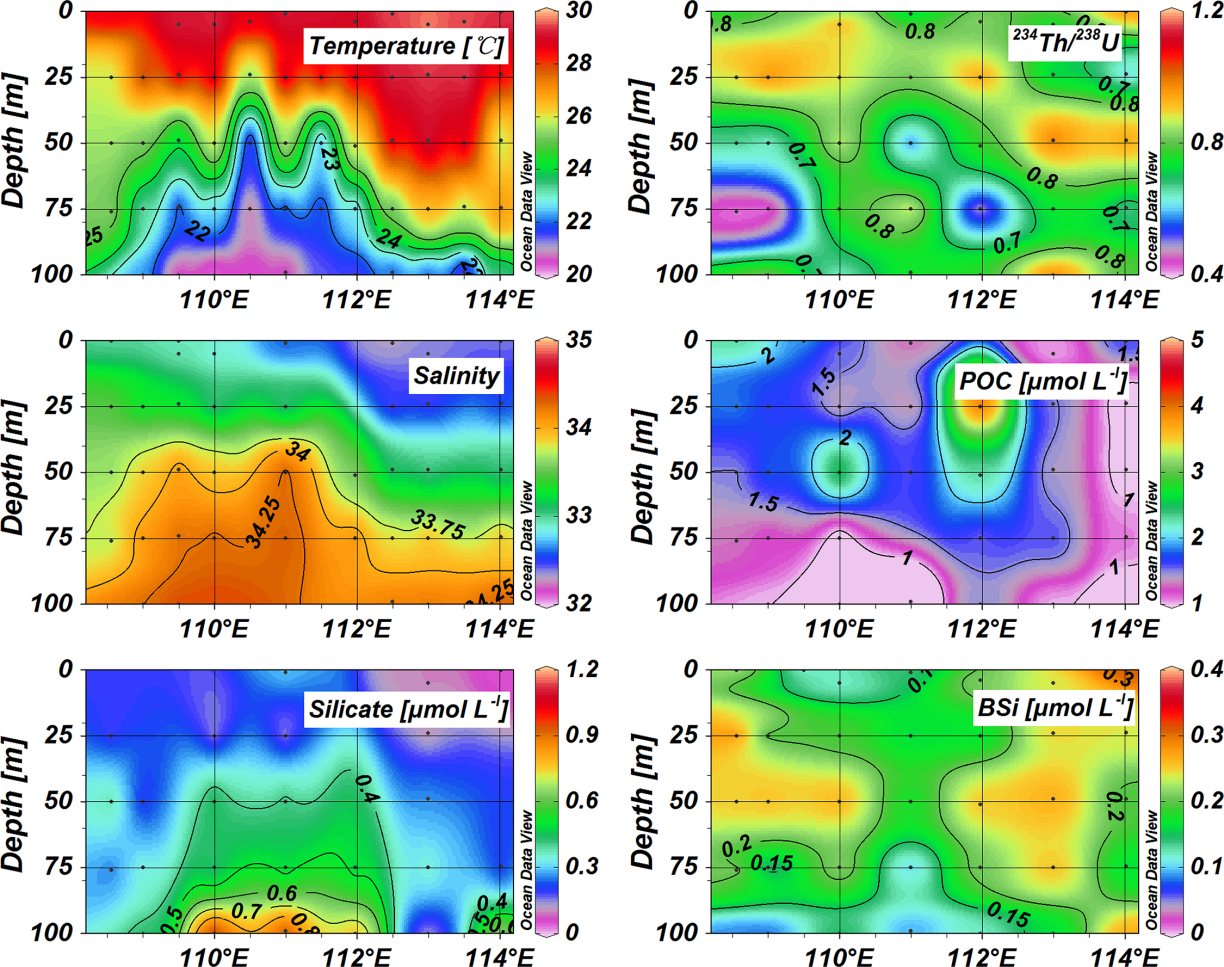
Vertical distribution of temperature, salinity, active silicate, POC, BSi, ^234^Th/^238^U ratios in the upper 100 m along Transect *I*.

**Fig 3:**
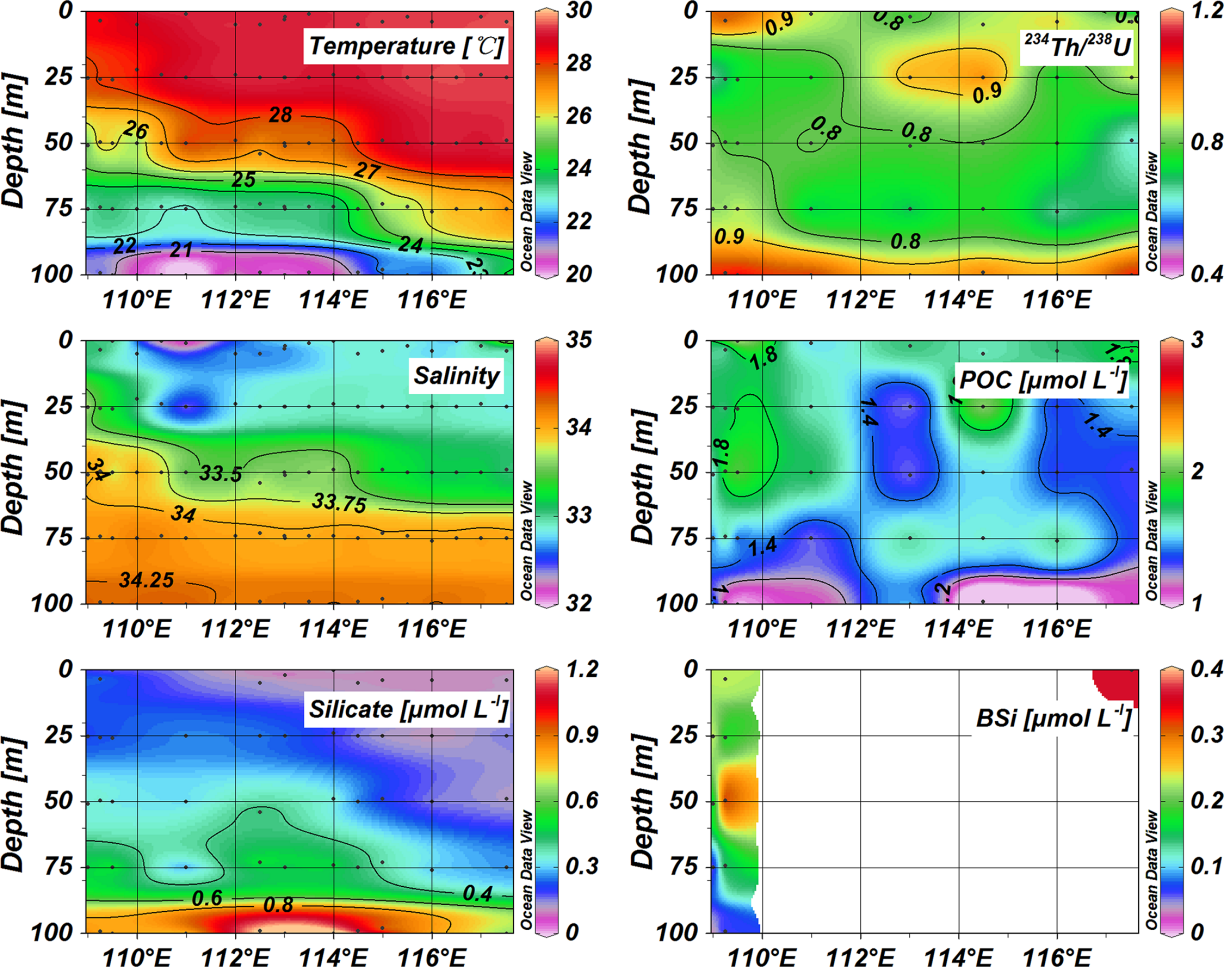
Vertical distribution of temperature, salinity, silicate, POC, BSi, and ^234^Th/^238^U ratios along Transect *II*.

**Fig 4:**
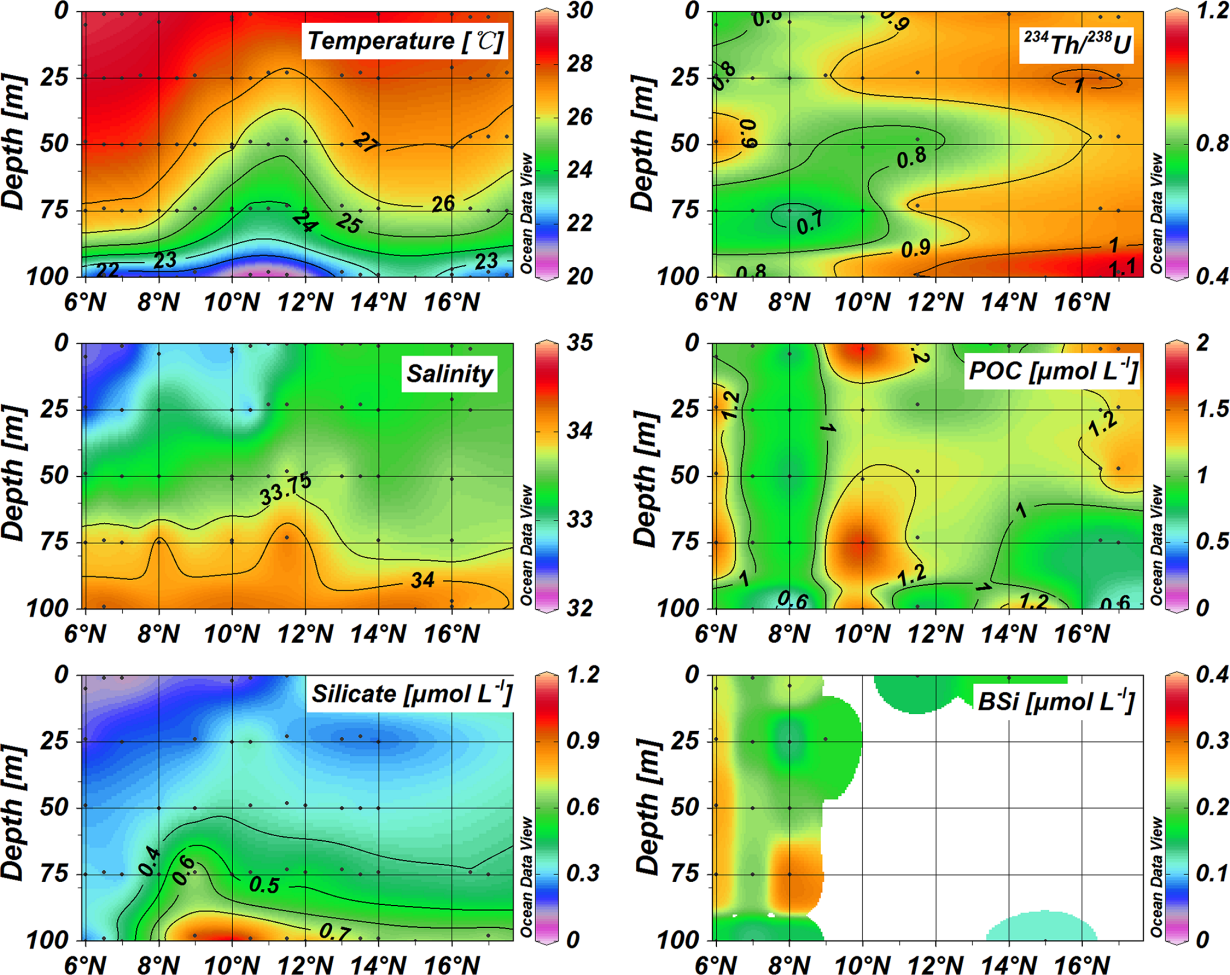
Distribution of temperature, salinity, active silicate, POC, BSi, and ^234^Th/^238^U ratios along Transect *III*.

Stations 33, 50, 52, 57, 58, 60 and 63 were located in the eddy, and were evidently influenced by the eddy on both 10 and 17 November. Stations 40, 42, 44, 46, 48, 54, 55, 72 and 75 were ordinary stations (Fig 1). Stations 36 and 69 were sampled on 17 November and, based on the SLA, they were regarded as outside stations. Since Stations 22 and 23 were located in the other eddy north to our studied eddy (Fig 1), they were not included in either the eddy or ordinary station groups. Such a separation was confirmed by the detailed distributions of hydrologic parameters and silicate concentration (Figs 2–4).

### Hydrologic parameters and silicate distributions

Temperature and salinity data are presented in Table 1, as well as the particulate components (i.e. POC and BSi) and the activity concentrations of ^234^Th. Along Transect *I*, the distribution of temperature and salinity showed the uplift of deep water introduced by the cyclonic eddy (Fig 2). Within the eddy, cold and salty water intruded into the euphotic zone. The mixed layer was compressed to less than 25 m based on the definition (Δ*T* = 0.8°C) for the mixed layer depth (MLD) [35], much shallower compared with around 50 m at outside stations. At the eddy center, temperatures were much lower than those of the surrounding water, while salinities were much higher. The vertical distribution of silicate concentrations also revealed the cyclonic eddy (Fig 2). In the upper 25 m, all waters showed low silicate concentrations. The silicate concentrations increased to around 0.5 μmol L^-1^ at 75 m at the eddy center; however, they were around 0.3 μmol L^-1^ at 75 m for the ordinary stations.

For Transect *II*, stations in the western area (including 57, 58, 60 and 63) showed cyclonic upwelling characteristics (Fig 3). The MLD was less than 25 m in the eddy, while it reached up to 50 m at other stations. Transect *III* also presented the cyclonic eddy at Station 33 (Fig 4). Cold water (T < 25°C) was observed in the upper 75 m around 11.5°N and high silicate concentrations revealed the intrusion of deep water into the upper 75 m.

### ^234^Th/^238^U disequilibria

The activity concentrations of total ^234^Th at all stations varied from 0.74 to 2.83 dpm L^-1^ (Table 1), which was comparable to the values obtained in spring [36]. In order to assess the difference between the eddy and ordinary stations, student’s *t*-test was used to check the two data groups in terms of a specific parameter as listed in Table 2. The sample sizes for statistical analysis are presented in parentheses in Table 2. No difference was observed for the ^234^Th deficits between the eddy and ordinary stations at the 95% confidence level. The ratios of particulate ^234^Th to dissolved ^234^Th, indicating its partitioning between particle and seawater, averaged 0.68±0.75 (n = 35) for the eddy and 0.74±0.74 (n = 53) for the ordinary stations (Table 2). Obviously, particles did not result in difference in the partitioning of ^234^Th. The average ^234^Th/^238^U ratio for all eddy stations was 0.85±0.20 (mean±sd, n = 35) and 0.79±0.18 (n = 53) for ordinary stations. Statistically, there was no difference between the eddy and outside stations (Table 2). Thus, the deficits of ^234^Th relative to ^238^U, via scavenging to particles and successive sinking, seemed to show little difference. Such a scenario indicated that the particle dynamics in terms of ^234^Th scavenging were similar in the studied eddy and ordinary water.

**Table 2:** Comparison of some parameters between the eddy and ordinary stations. E/O is the ratio of a specific parameter in the eddy to the surrounding water. The errors represent the standard deviation for data used to calculate the means.

Parameters	Outside^a^(mean±sd)	Eddy^a^(mean±sd)	E/O^b^(mean±sd)	Levene’s test*p*	*t*-test*p*
^234^Th/^238^U	0.79±0.18 (n = 53)	0.85±0.20 (n = 35)	1.0±0.3	0.488	0.168
^234^Th_P_/^234^Th_D_	0.74±0.74 (n = 53)	0.68±0.75 (n = 35)	1.0±1.6	0.597	0.714
F_Th_ (dpm m^-2^ d^-1^)	1218±419 (n = 11)	985±629 (n = 7)	0.8±0.6	0.054	0.356
POC (μmol L^-1^)	1.30±0.46 (n = 51)	1.42±0.56 (n = 34)	1.1±0.6	0.272	0.307
F_POC_ (mmol C m^-2^ d^-1^)	1.9±1.3 (n = 11)	1.5±1.4 (n = 7)	0.8±0.9	0.661	0.548
BSi (μmol L^-1^)	0.21±0.06 (n = 34)	0.17±0.09 (n = 20)	0.8±0.5	0.183	0.062
F_BSi_ (mmol Si m^-2^ d^-1^)	0.40±0.20 (n = 7)	0.18±0.15 (n = 4)	0.5±0.4	0.326	0.077
F_BSi_/F_POC_	0.21±0.13 (n = 7)	0.14±0.10 (n = 4)	0.7±0.6	0.685	0.384

^a^ The values in parentheses stand for the number of samples.

^b^ E and O refer to the eddy and ordinary stations. The *p* values were obtained from *t*-tests assuming *α* = 0.05.

### POC and BSi

For the eddy stations, the average POC concentration was 1.42±0.56 μmol L^-1^, comparable to that of 1.30±0.46 μmol L^-1^ at ordinary stations (Table 2). The average BSi concentration of 0.17±0.09 μmol L^-1^ in the eddy was also similar to that in the surrounding water which had a mean of 0.21±0.06 μmol L^-1^. Statistical analysis did not show any discernible difference in the BSi concentrations between the eddy and ordinary stations at the 95% confidence level. The average ratio of BSi concentrations in the eddy to those outside the eddy was 0.8±0.5.

## Discussion

### Eddy influence on BSi and POC abundance

Statistically, no difference in the POC concentrations was observed between the eddy and reference stations at the 95% confidence level (Table 2). The PP rates obtained using the ^14^C technique at the eddy stations 60 and 63 were 0.078 mmol C m^-3^ d^-1^ [37]. At ordinary stations (36, 69, 72 and 75), the PP rates ranged from 0.042 to 0.084 mmol C m^-3^ d^-1^ with a mean of 0.064±0.023 mmol C m^-3^ d^-1^ [37]. It seemed that there was little difference in the PP rates. Similarly, the eddy did not change the BSi abundance in the euphotic zone (Table 2). The comparable abundance of BSi in the present study was different from a few reports on an Atlantic mode-water eddy [4, 38], where increased BSi was observed within the eddy.

The eddy age would explain the difference in BSi variability between our study and the references. At the early life-stage, cyclones usually result in an increase of biogenic particles including POC and BSi [39]. However, at the decaying life-stage, a decaying biological response is reported, and even a negative NCP in an older cyclone in the Sargasso Sea [16]. In the present study, the temporal variability of SLA revealed that the eddy was in its decaying phase (Fig 1). Diatoms in the eddy probably did not show a discernible response to the eddy.

The δ^13^C signals, usually showing different values either for various phytoplankton species or for different PP rates in terms of specific species [40], also lends support to the small variability in the phytoplankton community and PP rates (Table 1). In the eddy, the δ^13^C values varied from -25.15 to -21.14‰, averaging -22.91±0.85‰ (mean±sd). At ordinary stations, the δ^13^C values ranged from -25.57 to -21.53‰ with an average of -23.22±1.00‰. Obviously, there was no difference, indicating little influence of the eddy on the PP at its decaying life-stage.

### Eddy influence on BSi and POC exports

The export fluxes of BSi and POC out of the euphotic zone were calculated using the ^234^Th-^238^U disequilibria. The mass balance of ^234^Th is expressed as [29, 41]:

$$\frac{dA_{Th}}{dt}=\lambda A_{U}-\lambda A_{Th}-P_{Th}+V$$ (1)

where *dA*_Th_/*dt* is the change rate of the total ^234^Th (i.e. non-steady state, NSS), *A*_Th_ and *A*_U_ are the total activities of ^234^Th and ^238^U, λ is the decay constant of ^234^Th (0.0288 d^-1^), *P*_Th_ denotes the net export flux of ^234^Th, and *V* is the sum of advection. In open oceans, the *V* term is usually minimal, as well as the NSS term [42].

In the present study, the influence of the NSS and *V* terms was evaluated. For the outside stations, the average activity concentration of ^234^Th was 1.91±0.14 dpm L^-1^. The difference between the average and ^234^Th activity at each station varied from -0.20±0.16 to 0.25±0.16 dpm L^-1^ in the euphotic zone. Together with the sampling dates for all outside stations, the estimated *dA*_Th_/*dt* values ranged from -34±27 to 42±27 dpm m^-2^ d^-1^. For the eddy stations, the NSS term corresponded to a range of -90±80 to 110±80 dpm m^-2^ d^-1^. On the one hand, the NSS term did not seem to evidently influence ^234^Th flux since it was around two orders of magnitude lower than the other terms, i.e. λ*A*_U_ and λ*A*_Th_. On the other hand, errors in the NSS term reached up to ~90%, indicating it was almost meaningless to our study. In general, at least 1–2 weeks between duplicate samplings are required to match the NSS model [42], and computing NSS terms with a sampling interval shorter than 10 days introduces massive errors [43]. Therefore, the NSS approximation was no better than just assuming an SS for short sampling intervals [42–43]. In the present study, sampling of the eddy stations was conducted within 4 d. Thus, the NSS term was not included in estimating the export fluxes of ^234^Th.

The advection term *V* consisted of vertical and horizontal advection, thus the export flux of ^234^Th could be calculated as:

$$P_{Th}=\lambda A_{U}-\lambda A_{Th}+\nu \frac{\partial A_{Th}}{\partial H}+\omega \frac{\partial A_{Th}}{\partial z}$$ (2)

where *v* is the horizontal current velocity, which was estimated based on the spatial current pattern as shown in Fig 1. The variable *ω* denotes the vertical mixing velocity. In the SCS, the vertical mixing velocity within a mesoscale scale eddy over a month timescale is around 3.4 m d^-1^ [44]. Considering the comparable timescale of the eddy in our study, this value was adopted. The term ∂*A*_Th_/∂*z* is the vertical gradient of ^234^Th activity from the twilight zone to the euphotic zone [45–46]. Here, it was estimated from the variability in the ^234^Th activity concentrations between 75 m and 200 m. The ∂*A*_Th_/∂_H_ term is the horizontal gradient of the ^234^Th activity based on its spatial pattern. The exports of BSi and POC were calculated using the proposed approach [29]:

$$P_{X}=P_{Th}\times \left(\frac{X}{{Th}_{P}}\right)$$ (3)

where *P*_x_ is the flux of particulate component “*X*” (i.e. BSi or POC) out of the euphotic zone, and (*X*/*Th*_*P*_)_z_ is the ratio of BSi (or POC) to particulate ^234^Th at the bottom of the euphotic zone.

For POC, both >53 and >1.0 μm particles were used to quantify the POC export. Because the >53 μm particle was not collected, the C/Th ratio in the >1.0 μm particle was used which was similar to the published POC calculation in the SCS [7, 36]. The relationship between the C/Th ratio and particle size is complicated [30–31]. Sometimes, the C/Th ratios in the >1.0 μm particle are higher than those in >53 μm particle [47–48], but this showed the opposite scenario in other studies [49–50]. A comparable C/Th ratio in both >53 and >1.0 μm particles is also observed [46, 51]. Although the influence of particle size on the C/Th ratio is unpredictable, below the euphotic zone the difference in the C/Th ratio between >53 and >1.0 μm particles is less than in the surface water [30–31]. In the SCS, the C/Th ratio is 1.4±0.1 μmol/dpm for >53 μm particles below the euphotic zone (100–125 m), and is 1.6±0.1 μmol/dpm for >1.0 μm particles [52]. This phenomenon is attributed to different POC in and below the euphotic zone. In the upper euphotic zone, the majority of POC were freshly produced by the plankton, while POC at the bottom of the euphotic zone might experience coagulation during sinking. Consequently, POC was large size, resulting in comparable C/Th ratios between >53 and >1.0 μm particles at the bottom of the euphotic zone. It should be noted that we cannot conclude that the POC export here was comparable to those from >53 μm particles although the reported C/Th ratios were similar between the >53 and >1.0 μm particles [52]. The influence of particle size on the POC flux is illustrated via a comparison between our results and others published for the SCS.

Besides particle size, the sampling technique also influences the POC/^234^Th ratio [30, 53]. A few studies suggest that bottle derived POC may overestimate the true POC concentrations owing to the adsorption of dissolved organic carbon (DOC) on the filter [54]. The increase of seawater volume may minimize the effect of DOC adsorption [36]. In the present study, POC was collected from 6–8 liters of seawater using a QMA filter with a diameter of 25 mm as in a previous study in the SCS [36], which corresponded to ~300–400 liters of seawater being pumped through a 142 mm diameter QMA filter. Such a strategy could decrease the influence of DOC. Indeed, the average of the POC/^234^Th ratios was 1.56±0.08 at the bottom of the euphotic zone in our study, consistent with that obtained using a 53 μm pore size Nitex screen in the SCS [52]. This ratio was also comparable to the sediment trap results in the lee of Hawaii (1.50±0.04 μmol dpm^-1^) [10]. Even though the POC/^234^Th ratio was comparable to those from the sediment trap or *in situ* pumps, the POC fluxes in the present study should be conservatively regarded as the upper limit reported in the SCS [7, 36].

We collected data related to the SCS to provide more information for comparison. Thus, the ^234^Th-derived POC flux, which ranged from 1.7 to 5.7 mmol C m^-2^ d^-1^ in November at 6°N [55], was comparable to our results; the sediment trap obtained POC export value at 720 m, which was 2.5 mmol C m^-2^ d^-1^ in November at a station (9°23′N, 113°14′E) [56] near Station 36, appeared to coincide with our result of 2.1±0.4 mmol C m^-2^ d^-1^ (Table 3); the ^234^Th- and ^210^Po-based average POC flux was 3.8±4.0 mmol C m^-2^ d^-1^ in April-May [36, 57]; and the POC export out of 100 m was 2.6 mmol C m^-2^ d^-1^ over a 4-yr timescale in the central and northern SCS [58]. These results indicated that the particle fluxes in our study were comparable with the published results.

**Table 3:** ^234^Th fluxes, POC/^234^Th and BSi/^234^Th ratios, and fluxes of POC and BSi out of the euphotic zone (100 m) in the tropical SCS.

Station	Depth	^234^Th flux	POC/^234^Th	BSi/^234^Th	POC flux	BSi flux
	(m)	(dpm m^-2^ d^-l^)	(μmol dpm^-1^)	(μmol dpm^-1^)	(mmolC m^-2^ d^-l^)	(mmolSi m^-2^ d^-l^)
KJ22	100	35±171	0.98±0.09	-	0.1±0.2	-
KJ23	100	146±172	0.89±0.08	-	0.2±0.2	-
KJ33	100	256±178	2.06±0.26	-	0.5±0.4	-
KJ40	100	1723±151	0.88±0.09	0.27±0.03	1.5±0.2	0.46±0.06
KJ42	100	1011±149	2.82±0.42	0.56±0.08	2.8±0.6	0.57±0.12
KJ44	100	1515±139	0.83±0.07	0.37±0.03	1.3±0.2	0.57±0.07
KJ46	100	939±166	0.77±0.06	0.12±0.01	0.7±0.1	0.12±0.02
KJ48	100	1268±161	3.64±0.49	0.29±0.04	4.6±0.9	0.37±0.07
KJ50	100	1620±146	0.55±0.05	0.10±0.01	0.9±0.1	0.16±0.02
KJ52	100	616±172	0.90±0.10	0.22±0.02	0.6±0.2	0.14±0.04
KJ54	100	1699±156	1.14±0.11	0.09±0.01	1.9±0.3	0.16±0.02
KJ55	75	1214±138	2.56±0.30	0.47±0.06	3.1±0.5	0.57±0.09
KJ57	100	1321±171	1.33±0.09	0.02±0.01	1.8±0.3	0.03±0.01
KJ58	100	1573±206	2.44±0.43	0.24±0.04	3.8±0.8	0.38±0.08
KJ60	100	145±224	1.64±0.20	-	0.2±0.4	-
KJ63	100	1362±175	1.99±0.15	-	2.7±0.4	-
KJ36	100	1259±215	1.66±0.19	-	2.1±0.4	-
KJ69	100	575±212	0.75±0.08	-	0.4±0.2	-
KJ72	100	1653±186	1.08±0.08	-	1.8±0.2	-
KJ75	100	545±192	0.99±0.09	-	0.5±0.2	-

The average ^234^Th flux was 985±629 dpm m^-2^ d^-1^ inside and 1218±419 dpm m^-2^ d^-1^ outside the eddy (Table 3). The average BSi flux in the eddy was 0.18±0.15 mmol Si m^-2^ d^-1^ (Table 3), comparable to 0.17±0.06 mmol Si m^-2^ d^-1^ observed in a mode-water eddy in the Sargasso Sea [4]. It was 0.40±0.20 mmol Si m^-2^ d^-1^ for the ordinary stations. Student’s *t*-test indicated no difference between the eddy and ordinary stations in terms of BSi fluxes at the 95% confidence level (*p* = 0.077, Table 2). This result lent support to the little influence of the studied eddy on BSi export in its decaying phase. This scenario was different from observations in cyclonic eddies at their early life-stages. For example, three to four times higher BSi flux is observed in an eddy in the subtropical North Pacific [3, 10, 59]. A two- to three-fold increase of the BSi flux is also found in a mature stage of an eddy in the Sargasso Sea [4]. Even up to ~20 times higher BSi export comparable to the Bermuda Atlantic Time-series Study site is observed in a mode-water eddy [38]. This difference between our results and published results suggested that the increase of BSi export occurred mainly at the early life-stage of the eddy.

The average POC flux was 1.5±1.4 mmol C m^-2^ d^-1^ within the eddy, and 1.9±1.3 mmol C m^-2^ d^-1^ in the surrounding waters respectively. Statistically, the eddy did not cause evident variability in the POC flux (Table 2). Similar results are found in mature eddies in the North Pacific [3, 10] and in the Sargasso Sea [4], indicating that these subtropical cyclones may not be effective in exporting POC to the mesopelagic zone [59]. Conversely, these studies reveal increased exports of BSi, implying that this eddy may act as a silica pump [3, 59]. However, our results illustrated little variation in either BSi or POC exports. Based on these observations, further research is needed to examine the influence of eddy life-stage on the decoupling of POC and BSi exports in the tropical SCS.
